# Symbiotic Legume Nodules Employ Both Rhizobial *Exo*- and *Endo*-Hydrogenases to Recycle Hydrogen Produced by Nitrogen Fixation

**DOI:** 10.1371/journal.pone.0012094

**Published:** 2010-08-10

**Authors:** Christopher O. Ciccolella, Nathan A. Raynard, John H-M. Mei, Derek C. Church, Robert A. Ludwig

**Affiliations:** Sinsheimer Laboratories, Department of Molecular, Cellular and Developmental Biology, University of California Santa Cruz, Santa Cruz, California, United States of America; University of Liverpool, United Kingdom

## Abstract

**Background:**

In symbiotic legume nodules, endosymbiotic rhizobia (bacteroids) fix atmospheric N_2_, an ATP-dependent catalytic process yielding stoichiometric ammonium and hydrogen gas (H_2_). While in most legume nodules this H_2_ is quantitatively evolved, which loss drains metabolic energy, certain bacteroid strains employ uptake hydrogenase activity and thus evolve little or no H_2_. Rather, endogenous H_2_ is efficiently respired at the expense of O_2_, driving oxidative phosphorylation, recouping ATP used for H_2_ production, and increasing the efficiency of symbiotic nodule N_2_ fixation. In many ensuing investigations since its discovery as a physiological process, bacteroid uptake hydrogenase activity has been presumed a single entity.

**Methodology/Principal Findings:**

*Azorhizobium caulinodans*, the nodule endosymbiont of *Sesbania rostrata* stems and roots, possesses both orthodox respiratory (*exo*-)hydrogenase and novel (*endo-*)hydrogenase activities. These two respiratory hydrogenases are structurally quite distinct and encoded by disparate, unlinked gene-sets. As shown here, in *S. rostrata* symbiotic nodules, haploid *A. caulinodans* bacteroids carrying single knockout alleles in either *exo*- or-*endo*-hydrogenase structural genes, like the wild-type parent, evolve no detectable H_2_ and thus are fully competent for endogenous H_2_ recycling. Whereas, nodules formed with *A. caulinodans exo*-, *endo*-hydrogenase double-mutants evolve endogenous H_2_ quantitatively and thus suffer complete loss of H_2_ recycling capability. More generally, from bioinformatic analyses, diazotrophic microaerophiles, including rhizobia, which respire H_2_ may carry both *exo*- and *endo*-hydrogenase gene-sets.

**Conclusions/Significance:**

In symbiotic *S. rostrata* nodules, *A. caulinodans* bacteroids can use either respiratory hydrogenase to recycle endogenous H_2_ produced by N_2_ fixation. Thus, H_2_ recycling by symbiotic legume nodules may involve multiple respiratory hydrogenases.

## Introduction

Legume root and stem nodules fix atmospheric dinitrogen (N_2_) yielding anabolic-N, which augments growth and reproduction of host plants. In these nodules, the biochemical conversion of N_2_ to ammonium is owed to endosymbiotic rhizobia (bacteroids) who carry the N_2_ fixation genes encoding the dinitrogenase complex. Whether N_2_ fixation occurs in legume nodules [1] or in pure cultures of diazotrophic (able to use N_2_ as sole N-source) bacteria [2], hydrogen gas (H_2_) is then co-produced. From subsequent mechanistic studies of dinitrogenase activity, H_2_ co-production is both stoichiometric and requires 2 ATP per H_2_ formed [3]–[4]. Yet in agronomic surveys, many legume nodules typically evolve H_2_ at high levels, and such H_2_ evolution rates correlate with N_2_ fixation rates [5]. However, in certain symbiotic legume nodules, bacteroids avidly fix N_2_ yet reproducibly evolve little or no H_2_ [1]. As this endogenous H_2_ production consumes metabolic energy, H_2_ recycling, which recoups that energy, allows increased efficiency of N_2_ fixation and, in principle, increased plant biomass yields [6]–[7]. This symbiotic nodule H_2_ recycling capability correlates with specific bacteroid strains, although host legume cultivars also contribute to H_2_ recycling and yield [8], [9]. Indeed, in biochemical assays, bacteroids isolated from H_2_ recycling (non-evolving) nodules show high levels of respiratory uptake hydrogenase activity [10], [11].

H_2_ recycling during N_2_ fixation was first observed with the aerobe *Azotobacter vinelandii*, a diazotroph but not a legume symbiont. In pure culture, *A. vinelandii* induces a particulate (respiratory) hydrogenase activity which oxidizes H_2_ at the expense of and tolerant of O_2_ [2]. In following studies with legume nodule bacteroids, such uptake hydrogenase activity was also affirmed [10]. In the ensuing forty years, hydrogenases, extensively studied, have proven both biochemically diverse and broadly distributed across bacteria and archaea [12]. Among aerobes and microaerophiles able to use H_2_ as energy source, uptake hydrogenase activities are typically classified as group I: heterodimeric, globular, hydrophilic proteins carrying a heteronuclear Ni,Fe-catalytic center; group I hydrogenases are generally O_2_ tolerant [12]. In cellular terms, the group I, Ni,Fe uptake hydrogenases are tightly associated with respiratory membranes via integral diheme *b-*type cytochromes, required for physiological activity [13], [14] [Bernhard]. As the group I cell membrane-peripheral complexes face the periplasm, or cell exterior [15], they may be termed *exo-*hydrogenases.

*Azorhizobium caulinodans*, a microaerophilic α-*proteobacterium* originally isolated as nodule endosymbiont of the host legume *Sesbania rostrata*, is capable of N_2_ fixation both *in planta* and in pure diazotrophic culture [16]. Recently, we discovered in *A. caulinodans* a second, novel respiratory hydrogenase encoded by the seven-gene *hyq* operon [17]. The inferred Hyq hydrogenase includes six different structural proteins, including a heterodimeric Ni,Fe-catalytic center hydrogenase conserved with group I enzymes. From bioinformatic analyses, the remaining four Hyq proteins are all membrane-integral [17]. Because all six Hyq hydrogenase subunits are NADH:quinone oxidoreductase (respiratory complex I) homologs [18], the Hyq complex is classified with the reversible group IV hydrogenases [12]. Given structural and functional homology to respiratory complex I [18], the Ni,Fe-catalytic center heterodimers of group IV complexes associated with respiratory membranes presumably face the cell-interior and thus may be termed *endo*-hydrogenases.

## Results

### In symbiotic legume nodules both *exo*- and *endo*-hydrogenases recycle H_2_ produced by N_2_ fixation

To assess physiological roles for both bacteroid Hup *exo*- and Hyq *endo-*hydrogenases in symbiotic legume nodules fixing N_2_ and recycling H_2_, *A. caulinodans* haploid derivatives carrying precise (to the nucleotide pair) in-frame deletions of *hup* and *hyq* structural genes encoding the conserved catalytic subunits of, respectively, *exo*- and *endo*-hydrogenases were constructed and verified by nucleotide sequencing of mutant loci [17]. Specifically, *A. caulinodans exo*-hydrogenase null mutants carried in-frame, precise, complete *hupSL* deletions; *endo-*hydrogenase null mutants comprised precise, complete *hyqRBCEFGI* operon deletions. As well, haploid recombinant double-mutants carrying both *exo*- and *endo*-hydrogenase null alleles were also constructed (Methods). Pure *A. caulinodans* cultures were used to inoculate both stems and roots of *S. rostrata* seedlings aseptically germinated and individually cultivated under N-limitation (Methods). In *S. rostrata*, symbiotic nodules are developmentally determinate, not meristematic. While both stem- and root-nodules subsequently developed on inoculated plants only, as they are invariably absent on uninoculated plants, stem nodules were chosen for further study. Three week-old and five week-old determinate stem nodules were excised from inoculated plants and individually tested for N_2_ fixation activity, assaying acetylene-dependent ethylene production by gas chromatography with flame ionization detection (Methods). Excised stem nodules all showed similar (±15%) high levels of acetylene reduction activity when normalized per fresh nodule biomass (Table 1). Accordingly, all *A. caulinodans* strains tested were assigned both nodulation-competent (Nod^+^) and N_2_ fixation-competent (Fix^+^) phenotypes.

**Table 1:** N_2_ fixation and H_2_ recycling in *S. rostrata–A. caulinodans* stem nodules.

A. *caulinodans* endosymbiont	Genotype	Phenotype	N_2_ fixation†	H_2_ evolved‡
61305R	57100 *nic5R*	(virtual) wild-type	31.0±0.4	0.30±0.05
66081	61305R *▵hupSL2*	*exo*-hydrogenase negative	33.0±0.4	0.32±0.05
66132	61305R *▵hyqRI7*	*endo*-hydrogenase negative	25.0±0.3	0.36±0.05
66204	61305R *▵hupSL2 ÄhyqRI7*	*exo*- and *endo*-hydrogenase neg.	25.0±0.3	27.0±0.3

† µmol (C_2_H_2_-dependent)C_2_H_4_ g^−1^ hr^−1^

‡ µmol H_2_ g^−1^ hr^−1^

**Figure 1:**
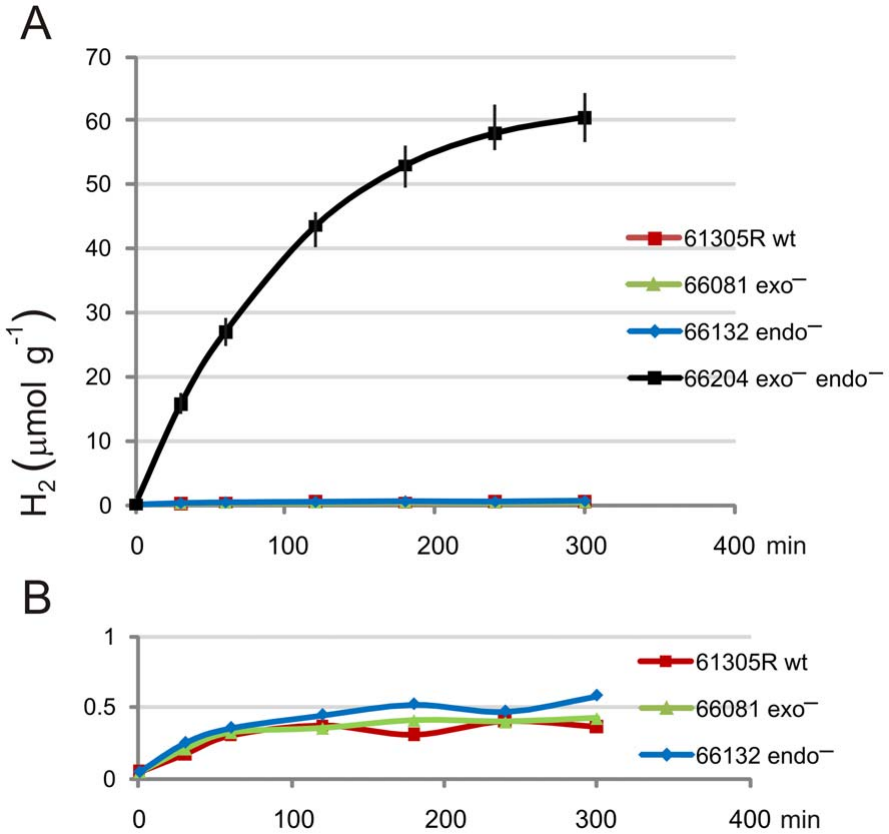
Hydrogen (H_2_) evolution by excised *S. rostrata* stem nodules elicited by indicated *A. caulinodans* strains as endosymbiont. (A) 70 µmol scale; (B) expanded ordinate, 1 µmol scale; evolved H_2_ measured as µmol g^−1^ (fresh biomass).

Additional excised nodules from these *S. rostrata* plants were simultaneously tested under air for H_2_ evolution activity using gas chromatography coupled to a reducing-compound photometric detector (Methods). In kinetic studies with excised nodules elicited by *A. caulinodans* strains 61305R (parental), 66081 (*exo*-hydrogenase mutant) and 66132 (*endo*-hydrogenase mutant), H_2_ evolution was nonexistent (Figs. 1a,b). Whereas, nodules elicited by double (*exo*- and *endo*-hydrogenase) mutant 66204 evolved H_2_ at very high rates (Fig. 1a) comparable to those measured for acetylene reduction (Table 1). Thus, H_2_ evolution by double-mutant 66204-elicited nodules was quantitatively owed to N_2_ fixation (dinitrogenase) activity. Results with five week-old determinate nodules from additional *S. rostrata* plants entirely corroborated results with three week-old nodules (data not presented). Pure bacterial cultures were reestablished from aseptically crushed nodules and strain identities verified by nucleotide sequencing of *hup* and *hyq* loci (Methods). In conclusion, *A. caulinodans* bacteroids in *S. rostrata* nodules employ both *exo*- and *endo-*hydrogenases to recycle endogenous H_2_ produced by N_2_ fixation. Moreover, H_2_ recycling is quantitative, entirely accounting for N_2_ fixation activities. Yet as measured by H_2_ evolution rates, bacteroid *exo*- and *endo*-hydrogenase are interchangeable and individually are fully competent to handle endogenous H_2_ recycling in symbiotic *S. rostrata* nodules.

### N_2_ fixing, microaerophilic α-proteobacteria able to recycle H_2_ carry *exo*- and *endo*-hydrogenase gene-sets

From bioinformatic analyses (Table 2), orthologous *hyq^+^* operons encoding *endo*-hydrogenase are generally present in N_2_ fixing microaerophiles able to recycle endogenous H_2_. These strains include both free-living diazotrophs as well as certain rhizobia, such as *B. japonicum,* the endosymbiont of *Glycine max* (soy). In *Rhizobium leguminosarum*, a metastable species with several descendant biovars each with genomes comprised of variable multipartite replicons, H_2_ recycling capability in symbiotic legume nodules varies among strains. As well, both the *hup* ^+^/hyp^+^ (*exo*-hydrogenase) and the *hyq* ^+^ (*endo*-hydrogenase) gene-sets are also variables [9], [19], [20]. Yet other diverse rhizobia (*e.g. Sinorhizobium meliloti* 1021; *Mesorhizobium loti* MAFF303099; *Rhizobium etli* CFN42; *Rhizobium sp*. NGR234) all incapable of H_2_ recycling in symbiotic legume nodules, completely lack both *hup* ^+^/hyp^+^ and *hyq* ^+^ gene-sets (Table 2). As the *hyq^+^* operon is also absent from anaerobic (fermentative) diazotrophs, fully aerobic diazotrophs (*e.g. Azotobacter spp*.), and non-diazotrophs generally, Hyq *endo*-hydrogenase seems co-selected with N_2_ fixation in microaerophilic (non-fermentative) α-proteobacteria. Nevertheless, in every N_2_ fixing microaerophile with both *exo*- and *endo*-hydrogenases, these gene-sets, as well the *nif* genes encoding N_2_ fixation activities are all unlinked (Table 2). Moreover, *A. caulinodans* haploid strains carrying complete (20-gene) *hup^+^/hyp^+^* (including *hupSL^+^*) operon deletions entirely lacking *exo*-hydrogenase and ancillary activities, nevertheless retain full H_2_ recycling activity both in pure cultures and in *S. rostrata* stem nodules. As well, *Rhodocista centenaria* (aka *Rhodospirillum centenum*) SW, which possesses the *hyq* ^+^ operon but not the *hup^+^/hyp^+^* operon (Table 2), completely recycles H_2_ in diazotrophic culture (data not presented). Accordingly, these *exo*- and *endo*-hydrogenase gene-sets seem fully autonomous.

**Table 2:** N_2_-fixing microaerophilic α-proteobacteria carrying orthologous *hup^+^/hyp^+^* (*exo-*hydrogenase) and *hyq^+^* (*endo*-hydrogenase) genes.

H_2_ recycling proficient:	legume host	diazotrophy	*hupSL^+^genes*	*hyq^+^ operon*
*Azorhizobium caulinodans* ORS571	*Sesbania rostrata*	+	AZC0598-0599	AZC4361-4355
*Beijerinckia indica* ATCC 9039	–	+	BIND1150-1151	BIND2473-2479
*Bradyrhizobium japonicum* USDA110	*Glycine max*	–	BLR1720-1721	BLR6338-6344
*Rhodocista centenaria* SW	–	+	–	RC11420-1415
*Rhodopseudomonas palustris* BisB5	–	+	RPD1162-1163	RPD3855-3850
*Xanthobacter autotrophicus* PY2	–	+	XAUT2173-2174	XAUT0165-0171
**H_2_ recycling deficient:**				
*Sinorhizobium meliloti* 1021	*Medicago sativa*	–	–	–
*Mesorhizobium loti* MAFF303099	*Lotus japonicus*	–	–	–
*Rhizobium etli* CFN42	*Phaseolus vulgaris*	–	–	–
*Rhizobium sp*. NGR234	*Vigna unguiculata*	–	–	–

## Discussion

Among legume-*Rhizobium* symbioses, H_2_ recycling was first reported in *Pisum sativum* (garden pea) nodules elicited by specific *Rhizobium leguminosarum* bv. *viciae* strains [2]. Genetic studies were subsequently undertaken with [*Brady*]*Rhizobium japonicum* strains able to recycle H_2_ in *Glycine max* (soy) nodules [21], [22]. Many subsequent studies with H_2_ recycling legume nodules all presumed uptake hydrogenase activity a single entity. These studies include combined genetic and physiological analyses which might have challenged this assertion. For the case of *A. caulinodans*, single mutants W58, U58 as well as *hupSL* impaired strain ORS571.2 all were reported to suffer substantial to complete loss of uptake hydrogenase activity [23], [24], [25]. Such conclusions are incompatible with the present finding: *A. caulinodans* employs two structurally and functionally distinct, genetically-independent, respiratory hydrogenases to recycle endogenous H_2_ produced by N_2_ fixation.

Whereas, early on the investigative timeline, *B. japonicum* single mutants unable to be cultured autotrophically on exogenous H_2_ yet still able to recycle endogenous H_2_ in soy nodules were identified [26]. As these strains showed induction of uptake hydrogenase activity in cultures shifted to O_2_ limitation (≤11 µ*M* DOT), they were perhaps understandably considered transcriptional control mutants hypersensitive to O_2_. With the benefit of hindsight, this phenotype is precisely that expected of true loss-of-function point mutants affecting *hup* operon structural genes encoding Hup *exo*-hydrogenase activity, were the observed limiting-DOT uptake hydrogenase activity in fact owed to Hyq *endo-*hydrogenase. In *A. caulinodans*, *hyq* operon expression requires NifA as transactivator [17], and the p*nifA^+^* promoter is in turn strongly transactivated by Fnr, which process requires physiological O_2_ limitation in diazotrophic culture [27]. In principle, both *exo*- and *endo*-hydrogenase gene-sets, despite being encoded at disparate loci in all organisms identified, might nevertheless share a common genetic predisposition, allowing strategic single mutations to convey dual loss-of-function. However, as strains carrying complete *hyq* operon deletions still possess wild-type Hup *exo-*hydrogenase activity, and *vice versa*, evidence for any genetic, post-transcriptional interaction or interdependence between the two gene-sets is entirely lacking.

As shown previously, in pure diazotrophic (N_2_ as sole N-source) cultures, *A. caulinodans exo-*hydrogenase knockout mutants grow as wild-type, whereas *endo*-hydrogenase knockout mutants exhibit slow growth [17]. Are *exo*- and *endo*-hydrogenase H_2_ recycling efficiencies in pure culture and in legume nodules then demonstrably different? Or, do diazotrophic phenotypes imply additional *endo-*hydrogenase function(s), *e.g.* chemiosmotic work associated with membrane ion translocation [28] not undertaken by *exo*-hydrogenase? Obviously, effective *exo*- and *endo-*hydrogenase cellular concentrations and/or distributions might be dissimilar in legume nodules and in pure diazotrophic cultures, even though both *hup^+^/hyp^+^* (*exo*-hydrogenase) and *hyq* ^+^ (*endo*-hydrogenase) gene-sets are then strongly transcribed [17], [25], [29]. Because *hup* mutants suffer loss of chemoautotrophy with exogenous H_2_ as energy substrate [17], [26], *exo-*hydrogenase kinetic behavior may constitute simple diffusion control. Because *hyq* mutants do not adversely impact chemoautotrophy with exogenous H_2_, *endo-*hydrogenase kinetic behavior might not constitute simple diffusion control. A critical test of this hypothesis is still lacking. Diazotrophic liquid batch cultures typically employ constant sparging with relatively high gas-phase exhaust rates (0.5 min^−1^), complicating kinetic behavior and analysis of cellular processes with gaseous substrate(s) subject to simple diffusion control. In such pure liquid diazotrophic batch cultures bacterial densities typically reach 10^8^ cc^−1^, whereas in determinate *S. rostrata* nodules, bacteroid densities approach 10^11^ cc^−1^, the latter obviously more conducive to endogenous H_2_ recycling under simple diffusion control. Notwithstanding, given their apparent co-selection in N_2_ fixing micoaerophilic α-proteobacteria capable of H_2_ recycling, *exo*- and *endo*-hydrogenases likely possess additional, distinctive functionalities yet to be elucidated.

## Methods

### Bacterial strains and culture conditions

*Azorhizobium caulinodans* ORS571 wild-type (strain 57100), originally isolated from *Sesbania rostrata* stem-nodules [16], was cultured as previously described [30]. As 57100 wild-type is a pyridine nucleotide auxotroph, to serve as ‘virtual’ wild-type, all experiments reported here employ *A. caulinodans* 61305R, a 57100 derivative carrying an IS*50*R insertion in the (catabolic) nicotinate hydroxylase structural gene. Precise, in-frame deletion mutants were constructed by a ‘crossover PCR’ method [31]. Haploid *exo*-hydrogenase knockout mutants each carry a *hup*Δ*SL2* allele in which the (upstream) *hupS* translation initiation codon is fused in-frame to a synthetic 21np linker sequence fused in-frame to the (downstream) *hupL* termination codon. Similarly, haploid *endo*-hydrogenase mutants each carry a *hyq*Δ*RI7* allele, in which the *hyqRBCEFGI* operon has been replaced by a deletion allele comprising the *hyqR* initiation codon fused in-frame to the 21np linker sequence fused in-frame to the *hyqI* termination codon. After gene replacement, haploid strains carrying deletion alleles were verified by PCR and DNA sequencing analyses [17].

### *Sesbania rostrata* nodulation tests

*S. rostrata* plants were germinated, cultivated aseptically, and stem inoculated with pure *A. caulinodans* strain cultures as described [16]. Either three or five weeks post-inoculation, stem nodules were detached, weighed, individually placed in septated vials. Dinitrogenase activity was assayed kinetically by acetylene reduction [32] and product ethylene was measured by gas chromatography with flame-ionization detection. H_2_ evolution was assayed kinetically and measured by gas chromatography with reducing compound photometer detection (RCP1; Peak Laboratories LLC, Mountain View, CA.), both at atmospheric pressure and 29°C [33]. Enzymatic activities are expressed per gram nodule fresh-biomass at 29°C.
